# Wilkie Syndrome and Pseudo-Nutcracker Syndrome a Rare Combination: Description of a Case

**DOI:** 10.7759/cureus.18612

**Published:** 2021-10-08

**Authors:** Renato Farina, Tiziana Vasile, Pietro V Foti, Isabella Pennisi, Antonio Basile

**Affiliations:** 1 Radiodiagnostic and Radiotherapy Unit, Department of Medical and Surgical Sciences and Advanced Technologies “GF Ingrassia”, Istituto di Catania, Catania, ITA

**Keywords:** nutckracker syndrome, wilkie syndrome, color-doppler ultrasound, cardiovascular abnormalities, ct (computed tomography) imaging

## Abstract

Wilkie's syndrome is a very rare vascular alteration caused by congenital or acquired reduction of space between the superior mesenteric artery (SMA) and aorta. In acquired form, it is caused by perivascular adipose tissue reduction due to rapid weight loss and, if symptomatic, causes postprandial vomiting and weight loss. The left renal vein (LRV) when it has a retro-aortic course can be compressed in aorto-vertebral space (AVS); if the stenosis is severe it can cause venous congestion symptoms with left flank pain, microhematuria, and thrombosis, this vascular alteration is known as a pseudo-nutcracker syndrome. The combination of Wilkie's and pseudo-nutcracker's syndrome (PNCS) is very rare and has not yet been described in the literature. We describe a case of a 62-year-old woman who presented symptoms and alterations typical of two syndromes.

## Introduction

Superior mesenteric artery syndrome (SMA) [1] is a very rare vascular alteration caused by congenital or acquired aortic mesenteric angle (AMA) reduction. The SMA in WS originates from the aorta with an acute angle, causing mesenteric aortic-space (MAS) reduction with consequent duodenum and/or left renal vein (LRV) compression. In acquired form reduction of AMA is caused by rapid weight loss which induces reduction of peri-vascular adipose tissue and is more frequent in anorexic or oncological subjects. There is a cut-off value (22°) of AMA and a cut-off value (8 mm) of aorto-mesenteric distance (AMD), below which duodenal compression occurs with consequent sub-occlusive crisis, postprandial vomiting, and nausea that promote weight loss [2]. MAS reduction can also cause LRV compression which, if hemodynamically significant, becomes symptomatic causing left flank pain, microhematuria, secondary varicocele, and thrombosis; this pathological condition is known as anterior nutcracker syndrome (ANCS) [3]. Osteophytes, aortic aneurysms, or surgical outcomes can narrow the aorto-vertebral space (AVS) causing compression of the left retroaortic renal vein [4], if compression is severe and symptomatic, provokes so-called pseudo-nutcracker syndrome (PNCS) [5]. PNCS manifests itself with LRV flow congestion as in the case of ANCS. Surgical treatment of WS is most practiced currently and generally consists in resection of the first duodenal loop and retro-vascular duodenum and anastomosis between duodenum and second duodenal loop which are placed anterior to SMA [6]. In this work, we describe a rare case of WS and PNCS combination.

## Case presentation

**Figure 1:**
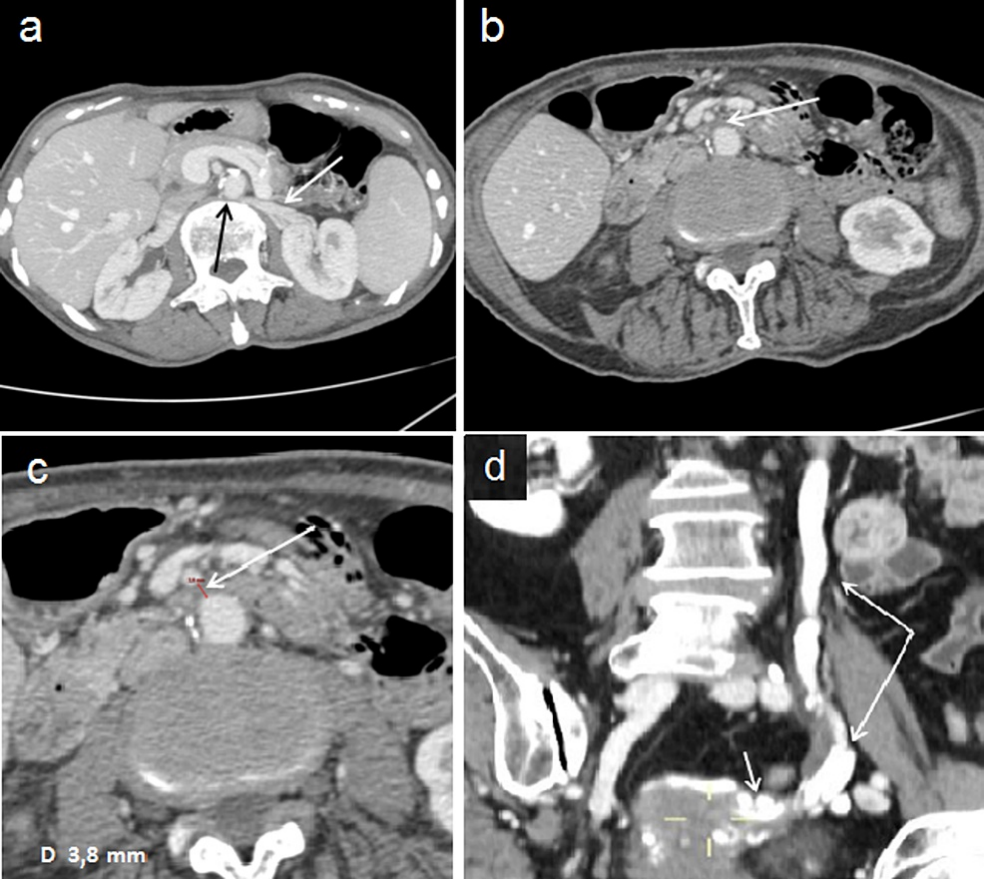
CT shows retroaortic LRV compression, duodenum compression, and left pelvic varicosities (a) A single 60-second post-contrast axial view shows LRV compression (black arrow) in aorto-vertebral space: LRV (with arrow). (b) A single 60-second post-contrast axial view shows duodenum (arrow) compression in aorto-mesenteric space. (c) In a 45-second post-contrast axial view aorto-mesenteric distance (arrow) appears reduced (6 mm). (d) A single 60-second post-contrast coronal view shows dilation of the left gonadal vein (long arrows) and ipsilateral gonadal plexus (short arrow).

A 62-year-old woman came to our observation for excessive weight loss (14.5: body mass index), recurrent postprandial vomiting, microhematuria, and pain in the left flank. The patient underwent computed tomography (CT) and Doppler US (DU). CT examination was performed with Optima 64 slice (GE-Healthcare, Chicago, IL) devise and ultrasound examination with a MyLab Nine (Esaote Biomedica, Genoa, Italy) devise, using a 3.5 MHz and 7.5 MHz probes. CT showed retroaortic LRV compression in AVS (Figure 1a), duodenum compression caused by reduced AMD (Figure 1b-1c), and left pelvic varicosities (Figure 1d).

**Figure 2:**
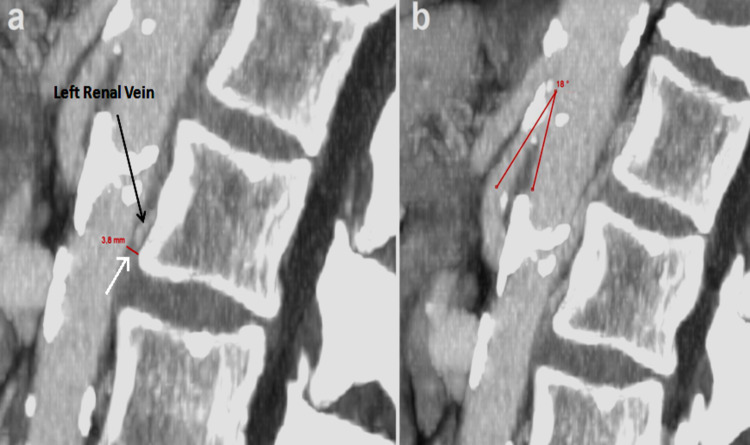
CT shows sagittal plane reconstructions (a) In sagittal view, it is possible to measure aorto-vertebral distance (arrow) which appears considerably reduced (3.8 mm). (b) In sagittal view, the aorto-mesenteric angle can be measured, which appears reduced (18 degrees), and superior mesenteric artery originating from aorta can be seen with an acute angle "beak sign."

Sagittal plane reconstructions showed an AVD of 3.8 mm and AMA of 18 degrees (Figure 2a-2b).

**Figure 3:**
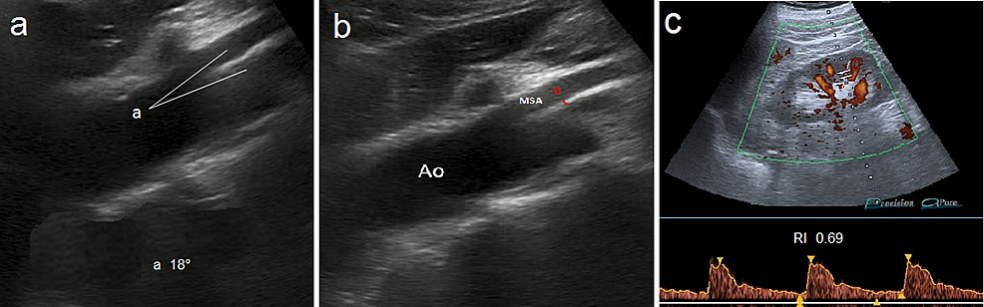
Images showing ultrasound examination (a) Longitudinal scan of abdominal aorta and MSA with aorto-mesenteric angle (a) measurement (18 degrees). (b) Aorto-mesenteric distance (a) measurement (6 mm). (c) Color Doppler US of left kidney shows regular Resistive Index (0.69).

Subsequently, the patient underwent an ultrasound examination which showed: AMA reduction (18 degrees) and AMD reduction (3.8 mm; Video 1 and Figure 3a-3b); LRV compression (stenotic tract diameter 3.5 mm); left varicocele (Sarteschi grade III); RI below maximum limits in the left kidney (0.69; Figure 3c); low PSV in LRV prestenotic tract (18.8 cm/sec); PSV increase in poststenotic tract (50.6 cm/sec) and in stenotic tract (78 cm/sec); LRV caliber reduction in stenotic tract (3.5 mm), prestenotic (8 mm) and poststenotic (7 mm) tract dilatation; FR was 2.69 (results are summarized in Table 1).

**Video 1:** B-mode ultrasound. Longitudinal scan of abdominal aorta showing aorto-mesenteric angle reduction.

**Table 1:** Summary of results obtained by duplex Doppler US PSV: peak speed velocity, LRV: left renal vein, RI: left kidney resistive index.

LRV	PSV	Caliber	FR	LRV RI
Flow ratio			2.69	0.69
Pre-stenotic tract	18.8 cm/s	8 mm		
Post-stenotic tract	50.6 cm/s	7 mm		
Stenotic tract	78 cm/s	3.5 mm		

The caliber and PSV of the right renal vein were 5.5 mm and 38 cm/s, respectively. Ultrasound was performed by an operator with 20 years of vascular ultrasound experience. Due to the patient-reported difficulty in digesting solid foods, we decided to recommend a high-calorie liquid diet for perivascular adipose tissue restoration and short distance (a month) monitoring with Doppler US. At the first control, the ultrasound showed an increase of AMA (20°), postprandial vomiting had decreased and his weight had increased by 3 kg.

## Discussion

**Figure 4:**
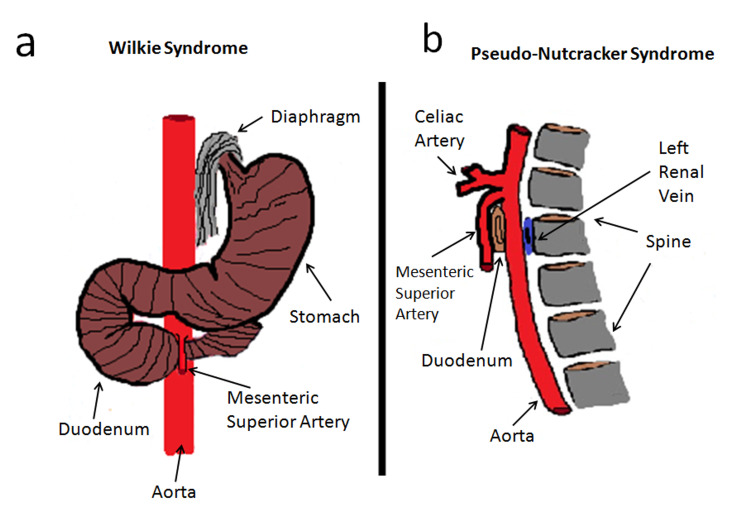
Scheme describing main anatomical structures involved in WS and PNCS (a) WS. Diagram according to the coronal plane, showing relationships between MSA, abdominal aorta, and duodenum. b: PNCS. Diagram according to the sagittal plane, showing relationships between the aorta, left renal vein, and spine.

The knowledge and accurate study of anatomical structures involved in WS (Figure 4a) and PNCS (Figure 4b) are fundamental for differential diagnosis.

The knowledge and accurate study of anatomical structures involved in WS (Figure 4a) and PNCS (Figure 4b) are fundamental for differential diagnosis. Although Doppler US is considered first-level examination for vascular compression syndromes study, in WS [7], it requires integration with level II examinations for duodenal compression evaluation. Since the presence of excess gas in the intestine prevents ultrasound, the duodenum study must be performed with conventional radiology (CR) or CT. Compared to CR, CT allows localized duodenal compression, AMA and AMD reduction, LRV compression and excludes other causes of compression; in sagittal view, the typical "beak sign" is highlighted, due to the acute angle that SMA forms with the aorta (Figure 3b) in ANCS. CT also showed a compression not elevated of LRV but sufficient to give varicocele. In PNCS, the aorto-vertebral distance must be measured in venous acquisition after 60 seconds after contrast administration of between the posterior wall of the aorta and anterior wall of the vertebral body at the point where LRV passes. The CT does not allow to carry out flowmeter investigations, which are very important in these cases to decide the most suitable treatment. The ultrasound measurements of PNCS must highlight the reduced distance between aorta and spine and the flow congestion in LRV upstream of compression; therefore with the patient in supine decubitus, scans must be performed according to the transverse plane that allows to identify the aorto-vertebral space and measure PSV and diameter of LRV before and after compression. In WS measurements of AMA must be performed with patients in supine decubitus with longitudinal scans of the abdominal aorta that allow visualization of AMS; the AMD must be measured 1 cm after the origin of SMA, using the software supplied with all ultrasound devices for angle measurement. While for WS there are cut-off values of AMA and AMD ​​for the diagnosis, for PNCS there are still no cut-off values in the literature ​​of AVD below which syndrome occurs. Duplex Doppler US allows us to obtain an estimate of LRV stenosis degree, thanks to FR calculation: an FR of 2.5 corresponds to stenosis of 50%, while in our case, it was slightly higher (2.69) and corresponded to stenosis of 53.8% which suggested that we adopt a conservative therapeutic approach. The most suitable method for the vascular study is intravascular ultrasound (IVUS), already used in May-Thurner Syndrome [8], which has shown an intraluminal characterization superior to angiography and which is increasingly expanding its diagnostic and, above all, interventional applications; however, its use is still limited by the availability of equipment and expert personnel. LRV compression can be mild and asymptomatic, without alterations in flow and renal function, in which case, it is known as "nutcracker phenomenon" [9] and does not require treatment. WS treatment must also be decided on the basis of duodenal compression degree and should, in our opinion, rely on a high-calorie diet to restore normal perivascular adipose tissue. In case of diet failure we prefer endovascular stenting, because it is less invasive than surgical treatment; endovascular stent allows to restore regular flow in LRV and, at the same time, to widen AMA and AVS [10]. In our case, the excessive narrowness of AVS did not guarantee, in our opinion, a good long-term stent seal, and as there are still few cases reported in the literature [11], we chose a prophylactic treatment with anticoagulant drugs to prevent LRV thrombosis.

## Conclusions

Combinations of multiple vascular compression syndromes are very rare and difficult to diagnose due to the rarity and non-specificity of the symptoms. On the other hand, asymptomatic vascular compressions are frequent and are often diagnosed randomly in routine CT and ultrasound examinations. The knowledge of these alterations can contribute considerably to the reduction of false negatives and allows to subject affected patients to periodic checks to evaluate their evolution. Failure to diagnose can have serious health consequences for those affected.

Vascular compression syndromes are poorly understood due to their rarity and combinations of multiple syndromes are even rarer. More widespread knowledge of these alterations can contribute considerably to the reduction of false negatives and thus avoid complications that can have serious consequences for the health of affected people. The purpose of this work is to contribute a greater knowledge of the information necessary to identify the subjects at risk to be subjected to periodic checks.
